# Cytoskeletal disruption-induced calcium dysregulation drives cell death in anti-IgLON5 disease

**DOI:** 10.1016/j.redox.2025.103854

**Published:** 2025-09-04

**Authors:** Lisanne Korn, Júlia Csatári, Andreas Schulte-Mecklenbeck, Laura Vinnenberg, Nadine Ritter, Paul Disse, Isabel Aymanns, Jan D. Lünemann, Catharina C. Gross, Petra Hundehege, Guiscard Seebohm, Heinz Wiendl, Thilo Kaehne, Matthias Pawlowski, Stjepana Kovac

**Affiliations:** a Department of Neurology with Institute of Translational Neurology, University Hospital Münster, Münster, Germany; b Department of Neurology, Medical Faculty, Heinrich-Heine University of Düsseldorf, Düsseldorf, Germany; c Institute for Genetics of Heart Diseases (IfGH), Department of Cardiovascular Medicine, University Hospital Münster, Münster, Germany; d Department of Drug Design and Pharmacology, University of Copenhagen, Copenhagen, Denmark; e Institute of Experimental Internal Medicine, Otto-von-Guericke University, Magdeburg, Germany

**Keywords:** IgLON5, AIE, Calcium dysregulation, Cytoskeletal disruption, Disease phenotype

## Abstract

Anti-IgLON5 disease is an autoimmune encephalitis with more chronic presentation including memory decline, sleep disorder, bulbar symptoms and movement disorder. Post-mortem brains of patients with anti-IgLON5 disease show neurodegeneration with tau deposition sparking interest in this ‘acquired tauopathy’ as a disease model for neurodegeneration, yet mechanisms of neurodegeneration remain unknown. Using a reductionist human iPSC-derived neuron-antibody model, we applied proteomics approach, electrophysiology and live cell imaging. iNeurons treated with anti-IgLON5 IgG presented with cytoskeletal disruption along with tau depositions, which correlated with endophenotypes. Accompanying calcium dysregulation was driven by impaired ER refill and mitochondrial dysfunction leading to cell death. Analogous cytoskeletal disruption is also reflected in the serum of treatment naïve patients using OLink proteomics. These findings provide insight into anti-IgLON5 disease pathology and pinpoint downstream signalling events of direct antibody-neuron interactions, which involve novel targets such as cytoskeletal disruption along with calcium dysregulation.

## Highlights

• IPSC-derived neuron-antibody disease model manifesting neurodegeneration after 7 days.
• INeurons treated with patient-derived IgG show cytoskeletal disruption.
• Patient IgG treated iNeurons exhibit Ca^2+^ dysregulation with impaired ER Ca^2+^ refill.
• Calcium dysregulation leads to mitochondrial dysfunction and cell death.
• Analogous cytoskeletal disruption in patient serum might serve as disease marker.

## Graphical abstract

**Figure:**
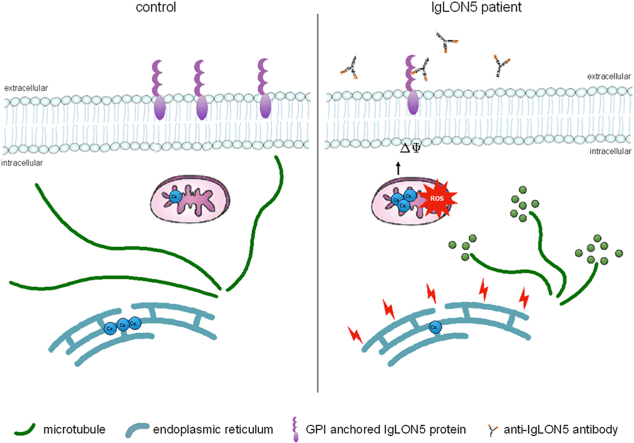

## 1 Introduction

Autoimmune encephalitides (AIEs) are associated with antibodies against neuronal or glial cells and are common, but underdiagnosed. A plethora of antibodies with intra- and extracellular targets has been described in recent years. Most extracellular targets are ion channels. More recently, neuronal cell surface proteins acting as cell adhesion molecules with particular expression at synaptic sites have been identified as targets in AIE [1]. Neurexin3 and IgLON5 proteins are such targets.

The IgLON protein family is a group of immunoglobulin (Ig) domain cell adhesion molecules consisting of five glycosylphosphatidylinositol (GPI) anchored proteins, which are involved in neuronal adhesion, neurogenesis and neuroplasticity [2]. IgLON5 keeps pre- and postsynaptic sides together and aligned, but more detailed information on the function of this IgLON isoform is lacking [3].

Anti-IgLON5 antibodies were detected in patients suffering from sleep problems, movement disorders, bulbar symptoms and cognitive decline. Anti-IgLON5 disease is an insidious disease, which has higher mortality rates and a higher likelihood of immunotherapy treatment failure than other types of AIEs [4], suggesting that irreversible degeneration occurs early in the disease course [5,6].

A study showing cytoskeleton disruption in a rodent anti-IgLON5 disease model suggests that antibody–neuron interaction triggers downstream signalling cascades underpinning the importance of timely intervention [7]. Further, there is a need for treatment targets which act beyond the initial antibody attack since disease progression occurs even after antibody removal by plasmapheresis in some patients [8]. We have previously shown that in AIE with antibodies targeting extracellular epitopes plasma cells - i.e. antibody producing cells - can be found in the cerebrospinal fluid (CSF) of patients, indicating that antibodies play a role in disease initiation and progression [9].

Post-mortem examination of patients’ brains showed hyperphosphorylated aggregations of the microtubule-associated protein tau in the tegmentum of the brainstem, hypothalamus and hippocampus [10]. Whether tau deposition itself is responsible for neurodegeneration in anti-IgLON5 disease or whether it serves as a marker of cytoskeletal disintegration remains unclear. A recent post-mortem study showed anti-IgLON5 immunoglobulin G (IgG)4 at predilection sites for tau pathology implicating that anti-IgLON5 antibodies precede the tau pathology. Interestingly, there was absence of complement activation in this post-mortem brain tissue, which argues against complement-mediated neurodegeneration and supports direct antibody effects [11].

Using a reductionist model of antibody-neuron interaction, we show that anti-IgLON5 IgG leads to cytoskeletal disruption, calcium dysregulation via impairment of calcium refill mechanisms, hypoexcitability and early cell death. Finally, this cytoskeletal disruption was also seen in serum of therapy naïve patients suffering from anti-IgLON5 disease, which could serve as a disease marker.

## 2 Methods

### 2.1 Cell culture and IgG isolation

The protocol for neural precursor cell (NPC) maintenance and differentiation into induced (i)Neurons was published before [12]. Subjects' consent was obtained according to the Declaration of Helsinki and this project has been approved by the ethical committee of the University of Münster (registration nos. 2013-350-f-S and 2016-053-f-S). For patients’ details see Supplementary Table 1. IgG fraction from serum and plasma samples from three IgLON5 patients and three healthy individuals was isolated with Protein G GraviTrap columns (GE28-9852-55, Cytiva). Isolated IgG fractions were dialysed overnight (887731, Thermo) to exchange the buffer to phosphate buffered saline (PBS) and after protein concentration (88527, Thermo) aliquots with a concentration of 1 mg/ml were stored at −80 °C until use. After 21 days of iNeuron differentiation, a final concentration of 75 μg/ml IgG fraction was added to the cells and refreshed with every media change for seven days. Experiments were conducted at 28 days after iNeuron induction.

### 2.2 Live cell imaging

Live cell imaging technique was explained before [12]. In short, experiments were conducted with an epifluorescence-inverted microscope and images were taken with a 40x oil objective. Dyes were diluted in artificial cerebrospinal fluid (120 mM NaCl, 2.5 mM KCl, 1.25 mM NaH_2_PO_4_, 22 mM NaHCO_3_, 25 mM glucose, 2 mM CaCl_2_, 2 mM MgSO_4_) and measured in the following settings.

**Table:** 

Dye	Manufacturer	Concentration	Excitation	Emission
Fura-2 AM	F1221 Invitrogen	5 μM	340 nm, 380 nm	510/80 nm
TMRM	T668 Invitrogen	40 nM	530 nm	705/72 nm
Hoechst/PI	H21492 Thermo, P4170 Sigma	5 μM	380 nm, 530 nm	510/80 nm, 705/72 nm
MitoSOX	M36008 Invitrogen	2.5 μM	530 nm	605 nm

Datasets were analysed with MetaFluor Fluorescence Ratio Imaging Software (Molecular Devices, LLC, Canada/US), Origin Version 2019 (OriginLab Corporation, Northampton, MA, USA) and ImageJ 1.54f (Wayne Rasband, National Institute of Health, USA).

### 2.3 Proteome analysis

*Normalisation:* Cells were lysed in 200 μl 8 M urea, 100 mM NH_4_HCO_3_, pH 8.0, and protein concentration was determined by BCA. Protein adjusted sample aliquots were subjected to sodium dodecyl sulfate–polyacrylamide gel electrophoresis (SDS-PAGE) to fine-adjust protein amounts for label free proteome analysis. After staining the gel with Coomassie Blue according to manufacturer's protocol the optical density of each sample lane was determined with a calibrated gel scanner in transmission mode and the relative protein amount was calculated.

*Digestion and fractionation:* Sample preparation for mass spectrometry was performed via in-solution digestion and strong cation exchange (SCX) fractionation. In brief, samples were four-fold diluted in 25 mM NH_4_HCO_3_, pH 8.0, and subsequently incubated with 5 mM dithiothreitol at room temperature for 1 h. Afterwards, reduced cysteine residues were carbamidomethylated via addition of 20 mM iodine acetamide at room temperature for 1 h. Proteins were digested by adding 2.5 μg trypsin (TrypsinGold, Promega, Madison, WI, USA) and incubated at room temperature over night. Digestion was stopped by adding formic acid (FAc) to a final concentration of 0.5 % and subsequently centrifuged at 15,000×*g* at 4 °C for 15 min. Resulting supernatant was subjected to a SCX column (SCX SpinTips, Protea Biosciences, Morgantown, USA) previously equilibrated with 60 μl acetonitrile (ACN) and washed with 0.1 % trifluoric acid (TFA). After sample application SCX column was washed with 60 μl 0.1 % TFA. Fractionation was achieved by stepwise elution with 60 μl of: 50 mM ammonium formiate, 20 % ACN, 0.5 % FAc; 75 mM ammonium formiate, 20 % ACN, 0.5 % FAc; 125 mM ammonium formiate, 20 % ACN, 0.5 % FAc; 200 mM ammonium formiate, 20 % ACN, 0.5 % FAc; 300 mM ammonium formiate, 20 % ACN, 0.5 % FAc and 5 % ammonium hydroxide, 80 % ACN. Eluted fractions were dried in a vacuum centrifuge.

*Mass spectrometry:* LC-MS/MS was performed on a hybrid dual pressure linear ion trap/orbitrap mass spectrometer (LTQ Orbitrap Velos Pro, Thermo Scientific, San Jose, CA, USA) equipped with an Ultimate 3000-nLC Ultra HPLC (Thermo Scientific, San Jose, CA, USA). Dried peptide fractions were dissolved in 10 μL 0.1 % TFA and subjected to a 200 cm μPACTM RP C18-csA column (PharmaFluidics, Ghent, Belgium). Separation was achieved by applying a gradient from 2 % ACN to 35 % ACN in 0.1 % formic acid (FA) over a 180 min gradient at a flow rate of 1.6 μL/min. The LTQ Orbitrap Velos Pro MS exclusively used CID-fragmentation when acquiring MS/MS spectra, consisting of an orbitrap full MS scan followed by up to 20 LTQ MS/MS experiments (TOP20) on the most abundant ions detected in the full MS scan. The essential MS settings were as follows: full MS (FTMS; resolution 60,000; *m/z* range 400–2000); MS/MS (Linear Trap; minimum signal threshold 500; isolation width 2 Da; dynamic exclusion time setting 30 s; singly charged ions were excluded from selection). Normalised collision energy was set to 35 %, and the activation time was set to 10 ms.

*Data processing:* Raw data processing and protein identification of the high resolution orbitrap datasets were performed with *de novo* sequencing algorithms of PEAKS Studio 8.0 (Bioinformatics Solutions Inc., Waterloo, Canada) using the SwissProt database. The false discovery rate was set to <1 %. Label free quantification (LFQ) was achieved by using PROGENESIS QI for proteomics (Nonlinear dynamics/Waters).

Raw data, complete identification listings and supplementary information are available via ProteomeExchange with identifier PXD052617.

### 2.4 Microelectrode array (MEA)

Field potentials of iNeuron activity were measured on 9-well MEA chips that were plasma-cleaned and coated with Matrigel (Corning) over night. After incubating the chips with FBS (Sigma Aldrich) at 37 °C for 30 min, cells were detached with TrypLE (Gibco) and plated onto the MEA chip for about 96 h. Due to the heterogeneity of neuronal signals, which are dependent on the density of iNeurons captured within the region of interest analysed by MEA, we performed longitudinal analyses in the same region of interest over a period of 7 days. A reference signal was recorded under basal conditions and then IgG incubation for controls and patients (75 μg/ml) was started. On day 2, 4 and 7 measurements in the same wells were repeated. All recordings were conducted at 37 °C. Datasets were analysed using Cardio2D software (Multi Channel Systems MCS GmbH, Reutlingen, Germany) and Origin v9.0 (OriginLab Corporation, Northampton, MA, USA).

### 2.5 Patch clamp recordings

At day 21 of differentiation, cells were singularised and reseeded onto Matrigel®-coated coverslips. After 5–7 days of antibody incubation, spontaneous activity was recorded between days 28 and 35 of differentiation. Whole-cell recordings were conducted using a standard patch-clamp setup with an EPC-10 amplifier and PatchMaster software (HEKA Electronics, Lamprecht, Germany). Recording pipettes were crafted from borosilicate glass (G150TF-10, Clark Electromedical Instruments, Pangbourne, UK), with typical electrode and series resistance values of approximately 6 MΩ and 8–20 MΩ, respectively. Spontaneous activity was recorded at −70 mV across 60 sweeps, each lasting 10 s, at room temperature. The solutions used for recordings were as follows: (1) Extracellular solution (in mM): NaCl 125, KCl 2.5, NaH_2_PO_4_ 1.25, HEPES 30, Glucose 10, MgSO_4_ 2, CaCl_2_ 2; pH = 7.35, ∼305 mOsm.(2) Intracellular solution (in mM): NaCl 10, CaMeSO_4_ 82, K-BAPTA 11, HEPES 10, KCl 1, TEA-Cl 15, 4-AP 5, QX-314-Cl 3.35, Phosphocreatin 15, MgCl_2_ 1, CaCl_2_ 1, Mg-ATP 3, Na-GTP 0.5; pH = 7.25, ∼295 mOsm.

### 2.6 Immunocytochemistry

To evaluate antibody binding to our iNeuron culture, cells were incubated with the isolated IgG fraction from controls or patients for 1 h at 37 °C. After fixing with 4 % PFA for 15 min at room temperature, secondary antibody Alexa Fluor 594 anti-human IgG (A-11012, Invitrogen, 1:500) was incubated for 1 h at room temperature.

For the analysis of tau and p-tau distribution, iNeurons were incubated with 75 μg/ml IgG fraction of either ctrl or patient for 7 days. Cells were fixed with 4 % PFA for 15 min at room temperature and subsequently blocked with PBS, 10 % goat serum and 0.3 % Triton-X for 1h at room temperature. Primary antibodies tau (MAPT, A0024, Dako, 1:500) and p-tau (AT8, MN1020, Invitrogen, 1:500) were diluted in PBS, 2 % goat serum and 0.1 % Triton-X and incubated at 4 °C over night. Secondary antibodies Alexa Fluor 594 (A-11012, Invitrogen, 1:1000) and Alexa Fluor 488 (A-11001, Invitrogen, 1:1000) were incubated for 1 h at room temperature. Coverslips were mounted in fluorescent mounting medium containing DAPI (00-4959-52, Invitrogen).

Imaging was conducted with a fluorescence microscope with an Apotome attachment (Axio Imager 2, Zeiss) equipped with a 63x or 100x oil objective and analysis was performed using ImageJ 1.54f (Wayne Rasband, National Institute of Health, USA).

### 2.7 OLink

Serum samples of healthy individuals and IgLON5 patients were cryo-conserved at −80 °C. Samples were shipped to OLink and investigated by proximity extension assay with next-generation sequencing (PEA-NGS). Resulting Ct values representing protein levels were log2 transformed and inverted to result in normalised protein expression (NPX) values where an increase by one corresponds with a doubling of protein concentration. Relative data from about 1500 proteins was filtered for proteins involved in microtubule and cytoskeleton related processes, and intracellular transport, respectively, by GO terms using the function “olink_pathway_enrichment” from the package OlinkAnalyze (v3.7.0). Sparse partial least squares discriminant analysis (sPLS-DA) was performed in R (4.3.0) using Rstudio (2023.06.1) by employing the mixOmics package (6.24.0). keepX was set to (10,10) with ncomp = 2. Receiver operating characteristic (ROC) analysis was performed using a composite score of linearised NPX data (2^NPX). Therefore, pROC package (1.18.4) was used in R with the plot.roc function.

### 2.8 IgG subclass composition

IgG subclass composition was determined in collaboration with EUROIMMUN Medizinische Labordiagnostika AG, Lübeck, Germany. Blood samples from patients and controls were incubated on a Mosaik 6 plate, which was coated with IgLON5. As secondary antibodies anti-IgG1-4 were used and semiquantitative analysis with a microscope was determined as titre. Analysis of IgG subclass composition in patient blood samples revealed that patient 1 and patient 3 predominantly had IgG1, while patient 2 exhibited a higher proportion of IgG4 (Supplementary Fig. 1A).

### 2.9 Multiplex analysis of complement activation

Concentrations of C5a, chemoattractant cleavage product of complement factor 5, and the soluble terminal complement complex SC5b9 were quantified in the supernatant of iNeuron cultures with and without the complement inhibitor cobra venom factor (CVF) using a *multiplex* ELISA based on chemiluminescence (Quidel, San Diego, USA, cat. number: A900). Data was obtained with Imager L from Quansys, using Q-View Software 3.11 for analysis. Multiplex analysis showed no differences in terminal complement component activation (Supplementary Fig. 1B), suggesting that effects observed are not due to complement activation but direct antibody-neuron interactions.

### 2.10 Statistical analysis

Statistical analysis for all experiments except proteome and OLink analysis was performed using GraphPad Prism v6.01 (GraphPad Software Inc., CA, USA). Statistical significance was tested for using one-way ANOVA and post-hoc Tukeys test. The significance level was ∗p < 0.05, ∗∗p < 0.01, ∗∗∗p < 0.001, ∗∗∗∗p < 0.0001.

## 3 Results

### 3.1 Anti-IgLON5 IgG impaired neuronal network function, glutamatergic signalling and led to cell death

**Fig. 1:**
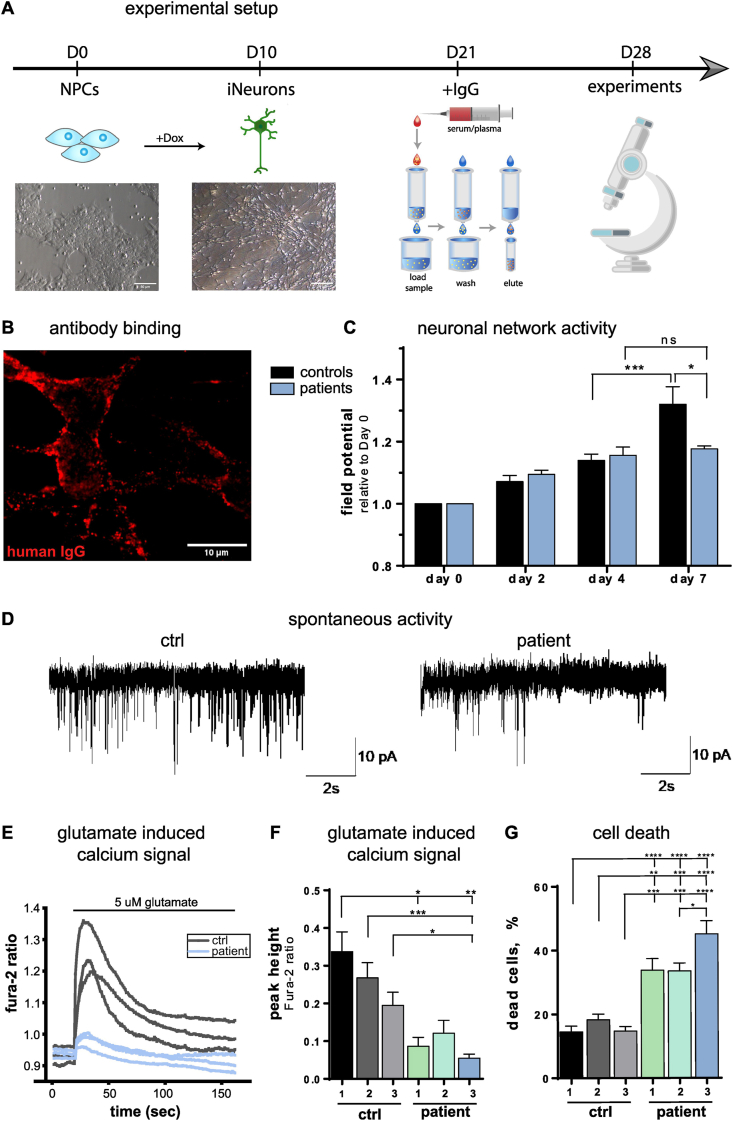
Antibody-induced neurodegeneration after 7 days of IgG incubation. (**A**) From day (D) 0 to D10, the forward programming protocol for generating cortical neurons from NPCs is applied and phase contrast images of NPCs (D0) and cortical neurons (D10 after induction) are shown. After 3 weeks of neuron maturation, IgG fraction is isolated from serum/plasma from IgLON5 patients and controls and incubated with the healthy induced neurons (iNeurons) for 7 days. At 28 days after inducing differentiation, experiments were performed. (**B**) Immunocytochemical staining of human IgG binding to the plasma membrane of iPSC-derived neurons incubated with IgLON5 anti-IgLON5 IgG. (**C**) MEA analysis measuring network activity after incubating iNeurons with IgG fractions of controls versus IgLON5 patients. (**D**) Exemplary traces of spontaneous activity of iNeurons either treated with control IgG or anti-IgLON5 IgG recorded via patch clamp. (**E**) Representative traces of calcium imaging with fura-2 of control and anti-IgLON5 IgG treated neurons after physiological glutamate stimulation (5 μM). (**F**) Peak amplitude of glutamate evoked calcium signal in anti-IgLON5 IgG compared to control IgG treated neurons. (**G**) Cell death assay with Hoechst (all cells) and propidium iodide (PI, dead cells) on neurons incubated with IgG fraction. Cell death was calculated by PI+/Hoechst + cells in percent.

We differentiated cortical neurons from human NPCs and incubated the induced (i)Neurons with the IgG fraction of either IgLON5 patients (anti-IgLON5 IgG) or healthy controls for 7 days (Fig. 1A). iNeurons showed membrane specific binding of IgG in our human *in vitro* model (Fig. 1B) as well as more specific anti-IgLON5 antibody binding in IgLON5 transfected HEK cells (Supplementary Fig. 2A). A previous study has shown that anti-IgLON5 antibody incubation reduces neuronal network activity after 20 days [13]. To capture the earliest time point of neuronal network exhaustion after antibody binding, we conducted microelectrode array (MEA) measurements at various time points in the same region of interest (baseline, 2, 4, and 7 days of IgG incubation). Field potentials increased over time as iNeurons matured and connected. However, incubation with anti-IgLON5 IgG led to a stagnation of activity increase after 7 days of treatment, indicating a trajectory of network activity in iNeurons incubated with anti-IgLON5 IgG (Fig. 1C). This was paralleled by less frequent spontaneous activity in patch clamp recordings of anti-IgLON5 IgG incubated iNeurons (Fig. 1D) and a decrease in glutamate-induced calcium transients as seen in live cell imaging (Fig. 1E and F), further supporting anti-IgLON5 antibodies’ effect on membrane excitability. Additionally, a notable increase in dead cells was measured compared to controls, indicating a significant impact of anti-IgLON5 antibodies on cell viability (Fig. 1G). Based on the observed effects on neuronal activity, calcium response, and cell death, we were able to reiterate antibody-mediated network hypoactivity and neurodegeneration *in vitro* within 7 days of incubation with full IgG.

### 3.2 Proteome of anti-IgLON5 IgG treated iNeurons clustered around cytoskeleton, mitochondria and endoplasmic reticulum

**Fig. 2:**
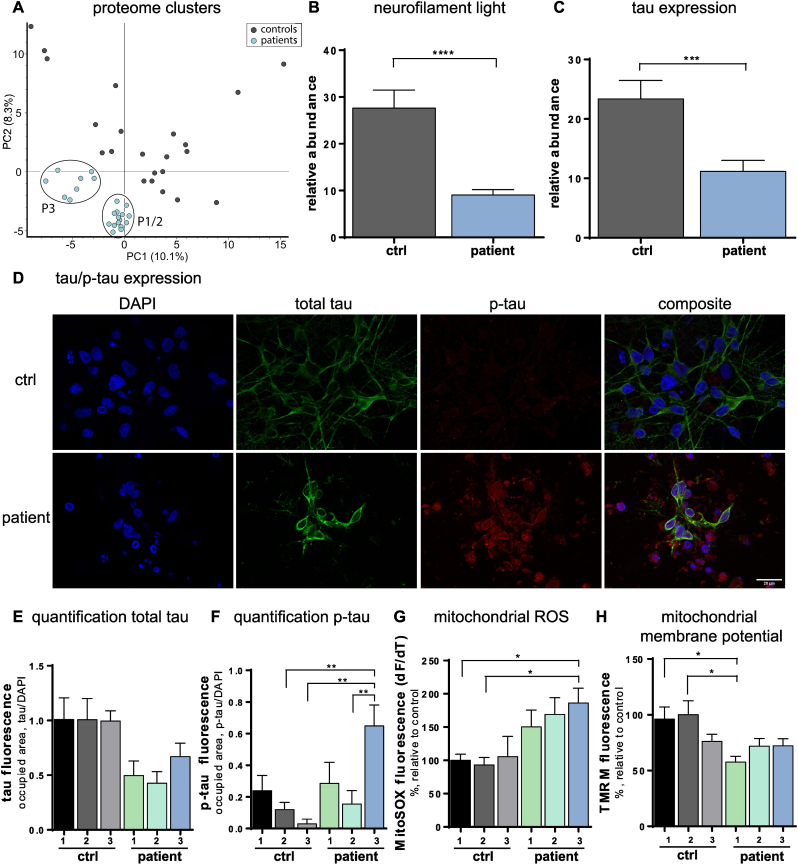
Mitochondrial dysfunction and cytoskeletal disruption in anti-IgLON5 IgG treated iNeurons. (A) Principal component analysis (PCA) shows significantly regulated proteins clustering together from anti-IgLON5 IgG treated iNeurons. (**B, C**) Quantification of proteome analysis shows the relative abundance of neurofilament light chain (**B**) and tau (**C**) in iNeurons incubated with either anti-IgLON5 IgG or control IgG. (**D**) Immunocytochemical staining of total tau (green), p-tau (red) and DAPI (blue). (**E, F**) Quantification of immunocytochemical stainings of total tau (**E**) and p-tau (**F**) measured as the occupied area normalised to DAPI. (**G**) Rate of mitochondrial ROS production measured with MitoSOX. (**H**) Mitochondrial membrane potential evaluated with tetramethylrhodamine (TMRM) fluorescence intensity.

We then performed an unbiased proteome analysis of iNeurons treated with anti-IgLON5 IgG in comparison to healthy control IgG to determine potentially affected pathways (Supplementary Fig. 3A). Principal component analysis (PCA) of proteome data revealed that significantly regulated proteins of patients 1 and 2 formed a distinct cluster, while patient 3 displayed a separate cluster, suggesting unique protein expression patterns among the patients and may indicate yet unknown proteome subtypes among IgLON5 patients (Fig. 2A). Differentially expressed proteins between anti-IgLON5 IgG incubated iNeurons and controls were analysed further, revealing clusters around cellular compartments such as cytoskeleton, mitochondria, and endoplasmic reticulum (ER) (Supplementary Fig. 3B–D), which were analysed in-depth.

### 3.3 Anti-IgLON5 IgG disrupted neuronal cytoskeleton and led to calcium dysregulation

Investigating cytoskeletal integrity, quantification of the proteomic data of neurofilament light chain (Fig. 2B) as well as the microtubule-associated protein tau (Fig. 2C) revealed that these structural proteins were less abundant in anti-IgLON5 IgG treated iNeurons compared to controls. Consistent with this, immunocytochemistry revealed that anti-IgLON5 IgG treated iNeurons exhibited a smaller area covered by tau (Fig. 2D and E), although this did not reach statistical significance when set in relation to DAPI, likely due to the high variance between differentiation batches. Phosphorylated tau (p-tau) deposition varied between the patients and notably, patient 3 - who already showed a distinct proteome cluster - displayed significantly increased levels of p-tau (Fig. 2D–F).

We hypothesised that cytoskeletal disruption leads to secondary mitochondrial and ER alterations. Mitochondria and the ER are the cellular hubs of calcium signalling [14] and thus reduced calcium signalling and glutamatergic network dysfunction (Fig. 1) may be explained by mitochondrial impairment and ER stress. In keeping with this hypothesis, higher rates of mitochondrial reactive oxygen species (ROS) production (Fig. 2G) and depolarised mitochondrial membrane potential (Fig. 2H) in anti-IgLON5 IgG treated iNeurons indicated notable alterations in mitochondrial function in response to anti-IgLON5 antibodies, in the absence of alterations in mitochondrial morphology (Supplementary Fig. 2B–D).

**Fig. 3:**
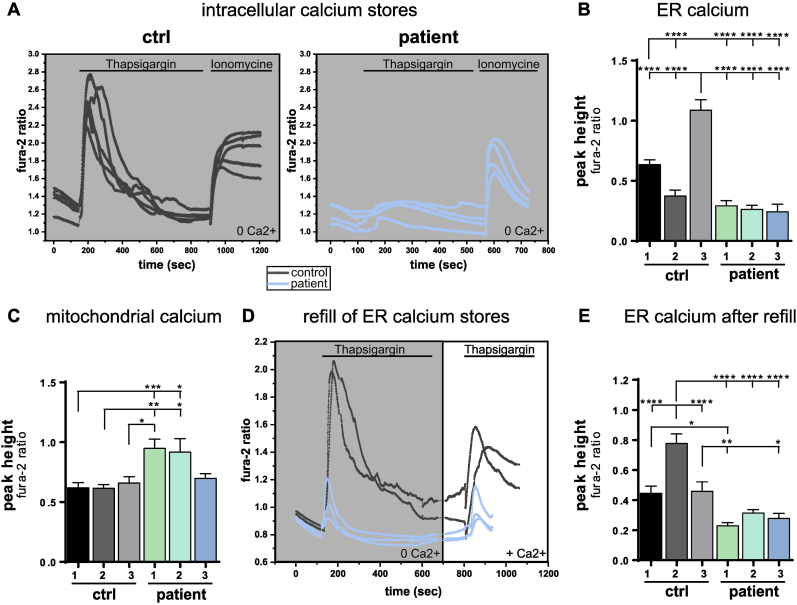
ER calcium mishandling in anti-IgLON5 IgG treated iNeurons. (**A**) Representative traces of intracellular calcium released from ER (thapsigargin) and mainly mitochondria (ionomycine) measured with fura-2 in a calcium-free environment. (**B–C**) Quantification of peak amplitude of ER calcium (released by thapsigargin) (**B**) and mitochondrial calcium (released by ionomycin) (**C**). (**D**) Representative traces of intracellular calcium released from ER (thapsigargin) in a calcium-free environment followed by adding extracellular calcium and finally releasing the refilled ER calcium again with thapsigargin. (**E**) Quantification of refilled ER calcium peak amplitude.

To assess intracellular calcium dynamics, calcium pools of ER and mitochondria were measured. Thapsigargin, an inhibitor of the sarcoplasmic/endoplasmic reticulum calcium ATPase (SERCA) pump, was added to deplete ER calcium. Subsequently, ionomycin, a calcium ionophore, was applied to release intracellular calcium stores, predominantly of mitochondrial origin due to prior ER calcium depletion (Fig. 3A).

Overall ER calcium pools were lower in cells treated with anti-IgLON5 IgG compared to controls (Fig. 3B). Patients 1 and 2 exhibited significantly higher mitochondrial calcium release whereas mitochondrial calcium levels in patient 3 were similar to iNeurons treated with control IgG (Fig. 3C). These results suggest altered calcium dynamics of iNeurons treated with anti-IgLON5 IgG. ER calcium refill capacity was evaluated to determine the cause of the decreased calcium levels. ER calcium was depleted with thapsigargin in calcium-free extracellular medium. After adding extracellular calcium, and allowing a few minutes of calcium uptake, ER calcium was released again (Fig. 3D). Peak height analysis after calcium refill revealed that controls exhibited significantly higher ER calcium release after refill compared to anti-IgLON5 IgG treated cells, indicating that controls have a greater capability for ER calcium refill (Fig. 3E). These findings suggest that anti-IgLON5 antibodies may impact the calcium homeostasis within the ER, further contributing to the functional differences observed in anti-IgLON5 IgG treated cells.

### 3.4 Cytoskeletal disruption can be seen in blood from IgLON5 patients

**Fig. 4:**
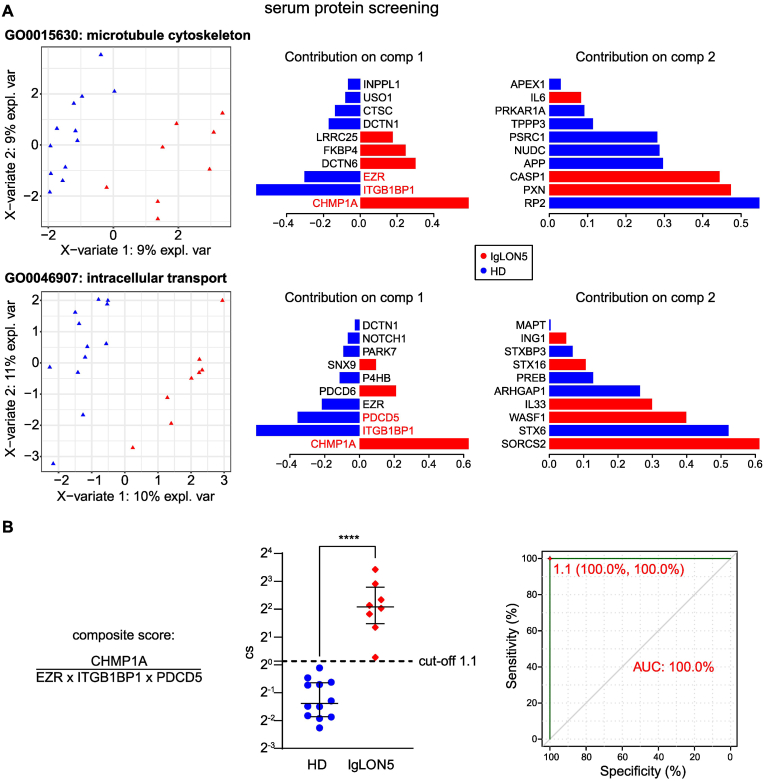
OLink proteomics in blood of IgLON5 patients compared to healthy controls. (**A**) Serum protein level analysis. Serum samples from 8 IgLON5 and 12 age-matched healthy individuals (HD) were investigated by OLink using proximity extension assay with next-generation sequencing (PEA-NGS). Proteins related to the GO-terms GO0015630 microtubule cytoskeleton and GO0046907 intracellular transport were selected and investigated by sparse partial least squares discriminant analysis (sPLS-DA) for their potential to separate both groups. The 10 best separating parameters for the first two axes were selected and their contribution was plotted. (**B**) The proteins most consistently separating both groups, IgLON5 patients and healthy donors (HD), were combined in a composite score. Therefore, normalised protein expression (NPX) levels were linearised and proteins increased in IgLON5 patients (CHMP1A) were divided by decreased ones (EZR, ITGB1BP1, PDCD5). Receiver operating characteristic (ROC) analysis identified a cut-off of 1.1 separating IgLON5 patients from HD with an AUC of 100 % (specificity 100 %, sensitivity 100 %). Statistical analysis was performed by Mann-Whitney test, ∗∗∗∗p < 0.0001.

Although IgLON5 protein is mostly expressed in the brain and testis, expression has been observed in multiple organs amongst others myoblasts [15]. We hypothesised that cytoskeletal disintegration as seen in the central nervous system (CNS) may also be observed in the blood of treatment naïve IgLON5 patients, even in the absence of a tissue phenotype other than CNS affection. Therefore, we investigated serum of eight therapy naïve patients (including the three patients investigated in our *in vitro* analysis) in comparison to twelve healthy donors (HD) by multiplex proteomic assay (OLink analysis). Both groups were clearly separated by sparse partial least squares discriminant analysis (sPLS-DA) by focussing on proteins related to microtubule cytoskeleton and intracellular transport dysfunction (Fig. 4A), suggesting differential abundance of associated proteins. The Top 20 proteins contributing to the separation of IgLON5 patients and HD were plotted individually (Supplementary Fig. 4). The most consistently differentially abundant proteins (Ezrin (EZR), Programmed cell death protein 5 (PDCD5), Integrin beta-1-binding protein 1 (ITGB1BP1) and Charged multivesicular body protein 1a (CHMP1A)) were combined in a composite score to increase robustness, sensitivity, and feasibility in separating both groups (Fig. 4B left), as exemplified before [16]. Receiver operating characteristic (ROC) analysis identified a cut-off of 1.1 resulting in a good sensitivity and specificity for the separation of IgLON5 patients from HD in this exploratory cohort (Fig. 4B right). These results show that the pathways identified *in vitro* are paralleled by changes in the blood presumably through affection of other tissues at a smaller scale.

## 4 Discussion

We here show that cytoskeletal disruption with changes in tau lead to neurodegeneration in a human iNeuron anti-IgLON5 disease model. In addition, iNeurons incubated with anti-IgLON5 IgG showed calcium dysregulation due to a weaker ER calcium refill capacity and functional mitochondrial impairment. Cytoskeletal disruption was also observed in the serum of untreated IgLON5 patients, suggesting its potential as a biomarker.

Anti-IgLON5 disease is a rare disease, but mechanisms involved in neurodegeneration in anti-IgLON5 disease may elucidate broad mechanisms of neurodegeneration in other more frequent forms of neurodegeneration such as Alzheimer's disease and frontotemporal lobar degeneration (FTLD). Interestingly, hyperexcitability has recently been recognised as an important driver in neurodegeneration such as is seen in Alzheimer's disease and amyotrophic lateral sclerosis [17,18]. We have previously shown that in monogenetic tauopathies (FTLD-MAPT) hyperexcitability may contribute to neurodegeneration [12] and in a study by Askin et al. hyperexcitability was seen in minutes to hours after anti-IgLON5 antibody-neuron contact and was able to induce tau pathology [19]. Surprisingly, in-line with a recently published study [13] we show here that rather than hyperexcitability impairment of glutamatergic signalling and excitability can be seen in anti-IgLON5 disease 7 days after the neuron-antibody contact suggesting that hyper- and hypoexcitability can occur at different stages of disease.

Using an unbiased proteome approach, we show that differences in proteome composition between anti-IgLON5 IgG treated iNeurons and controls cluster around cytoskeletal, ER and mitochondrial proteins. Interestingly, we found a distinct proteome cluster in patient 3. This patient was younger at disease onset and presented without a sleep disorder, which were identified as contributing factors to more tau deposition [20]. Notably, patient 3 was the only patient that exhibited significantly higher levels of p-tau compared to controls underlining autopsy studies that did not find tauopathies in all patients [11]. Disintegration of the cytoskeleton along with changes in the intracellular calcium homeostasis mainly driven by defective ER calcium refill occur, which are likely driving hypoexcitability and neurodegeneration in our *in vitro* disease model. While cytoskeletal disruption was identified by proteomic analysis and qualitatively supported by immunocytochemistry of tau, the quantification of tau staining did not reach significance likely due to variability in cell culture. Although our proteomic findings are not complemented by an additional verified supportive experiment, they are consistent with previous independent reports of cytoskeletal disintegration in the disease context [7].

The maintenance of ER calcium levels is essential for sustained intracellular calcium signalling. Disruption of this balance or accumulation of unfolded proteins causes ER stress, activating the unfolded protein response (UPR), which aims to restore ER function or, if necessary, triggers cell death [21]. During 10.13039/501100008680UPR, enhanced calcium transfer from the ER to mitochondria boosts adenosine triphosphate (ATP) production and supports protein folding, thus helping to alleviate ER stress [22]. However, excessive mitochondrial calcium uptake can cause mitochondrial permeability transition pore (mPTP) opening, leading to mitochondrial swelling, apoptosis, loss of oxidative phosphorylation, and increased ROS generation [23]. Calcium overload also disrupts mitochondrial membrane potential [24]. In our anti-IgLON5 IgG treated iNeurons, we observed elevated mitochondrial calcium, depolarised mitochondrial membrane potential, and significantly higher mitochondrial ROS levels versus controls, suggesting ER stress involvement. Targeting the ER-mitochondria calcium axis could therefore represent a promising therapeutic approach. Notably, tau phosphorylation alone can activate UPR, and pharmacologically induced ER stress may also promote tau hyperphosphorylation [25]. This suggests a potential positive feedback loop between UPR activation and tau pathology, which may drive neuronal degeneration. Since we detected hyperphosphorylated tau in our iNeurons - a feature also commonly found in post-mortem samples from patients [10] - these findings highlight a possible role for UPR in the development and progression of anti-IgLON5 AIE. Studying the effect of UPR blocking on IgLON5 pathology is thus intriguing and should be explored in future studies.

A short disease duration (median 1.25 years) was reported in three patients who showed no tau deposition [11]. Whether tau pathology would have emerged at a later stage in these individuals remains unclear. In our disease model, healthy iNeurons were exposed to IgLON5 antibodies derived from three different patients under identical experimental conditions. Nevertheless, the results varied markedly among the patients. No significant p-tau accumulation was observed in patients 1 and 2, whereas patient 3 exhibited a substantial increase in p-tau levels. Moreover, proteomic analysis revealed a distinct cluster of significantly regulated proteins in patient 3 that clearly separated from the clusters of patients 1 and 2.

These findings indicate that the pathological effects of anti-IgLON5 antibodies can differ significantly among patients. These subtypes may require different therapeutic approaches, potentially explaining why some patients do not benefit from immunotherapy [6]. However, our *in vitro* study involved a very small cohort and employed a reductionist model lacking immune cells. Therefore, larger multicenter studies are needed to explore the interplay between immune cell involvement, tau deposition, and treatment response.

The role of anti-IgLON5 antibodies as a primary or secondary event in disease pathogenesis has been debated. However, our study, alongside others, supports their direct pathogenic role [7,13]. In line with previous studies [6,8], all our patients predominantly had autoantibodies belonging to both the IgG1 and IgG4 subclasses. The relative pathogenic contribution of each subclass remains unclear. Since both are present in patients, it is crucial to determine whether they act independently or synergistically in driving the disease. Due to the limited availability of patient material, we were unable to purify the individual subclasses or analyse their effects separately. This underscores the need for future studies that address this question more directly through functional approaches. The absence of such data likely limits the interpretation of our findings and may obscure the potentially significant role of specific IgG subclasses in neuronal pathology induced by anti-IgLON5 antibodies.

In other autoimmune diseases, IgG4 antibodies are known to disrupt protein-protein interactions, thereby affecting signal transduction and cell adhesion [26]. Furthermore, such conditions often follow a gradual and sometimes fatal course [27] - features also observed in anti-IgLON5 disease, underscoring a potentially critical role for IgG4. Landa et al. (2023) demonstrated that anti-IgLON5 antibodies interfere with IgLON5 protein interactions, an effect mediated not only by IgG4 but also, unexpectedly, by IgG1 [30]. Moreover, IgG1 antibodies were found to induce irreversible internalisation of IgLON5 in cell culture models [5], and it was shown that they are able to activate the complement system [28].

While we recently demonstrated complement activation in anti-glutamate decarboxylase (GAD) AIE [29], complement deposits were absent in post-mortem analyses of IgLON5 patients [11]. We ensured that our *in vitro* model was serum-free to exclude complement activation, confirming that the observed neurodegenerative effects were caused by direct interactions between the antibodies and the iNeurons. This suggests that complement-mediated mechanisms may not play a major role in anti-IgLON5 pathogenesis.

Finally, we were able to show that also in the blood of IgLON5 patients pathways related to microtubules and intracellular transport are dysregulated. This dysregulation was strong enough to distinguish serologic profiles of IgLON5 patients from HD highlighting potential biomarkers of disease.

## CRediT authorship contribution statement

**Lisanne Korn:** Writing – original draft, Visualization, Investigation, Formal analysis, Data curation, Conceptualization. **Júlia Csatári:** Formal analysis, Data curation. **Andreas Schulte-Mecklenbeck:** Visualization, Formal analysis, Data curation. **Laura Vinnenberg:** Formal analysis, Data curation. **Nadine Ritter:** Formal analysis, Data curation. **Paul Disse:** Formal analysis, Data curation. **Isabel Aymanns:** Formal analysis, Data curation. **Jan D. Lünemann:** Supervision. **Catharina C. Gross:** Supervision. **Petra Hundehege:** Supervision. **Guiscard Seebohm:** Supervision. **Heinz Wiendl:** Supervision, Resources. **Thilo Kaehne:** Visualization, Supervision, Formal analysis, Data curation. **Matthias Pawlowski:** Writing – review & editing, Supervision, Conceptualization. **Stjepana Kovac:** Writing – review & editing, Writing – original draft, Supervision, Resources, Project administration, Funding acquisition, Conceptualization.

## Declaration of competing interest

Nothing to report.

## Data Availability

Proteome data are available at ProteomeExchange with identifier PXD052617.
