# Supplementary material for: Cytoskeletal disruption-induced calcium dysregulation drives cell death in anti-IgLON5 disease
Source: Redox Biol. 2025 Sep 4;86:103854. doi: 10.1016/j.redox.2025.103854 (PMC12454665; doi:10.1016/j.redox.2025.103854)
Supplement: Multimedia component 1 [file mmc1.docx]

**Supplementary Figures
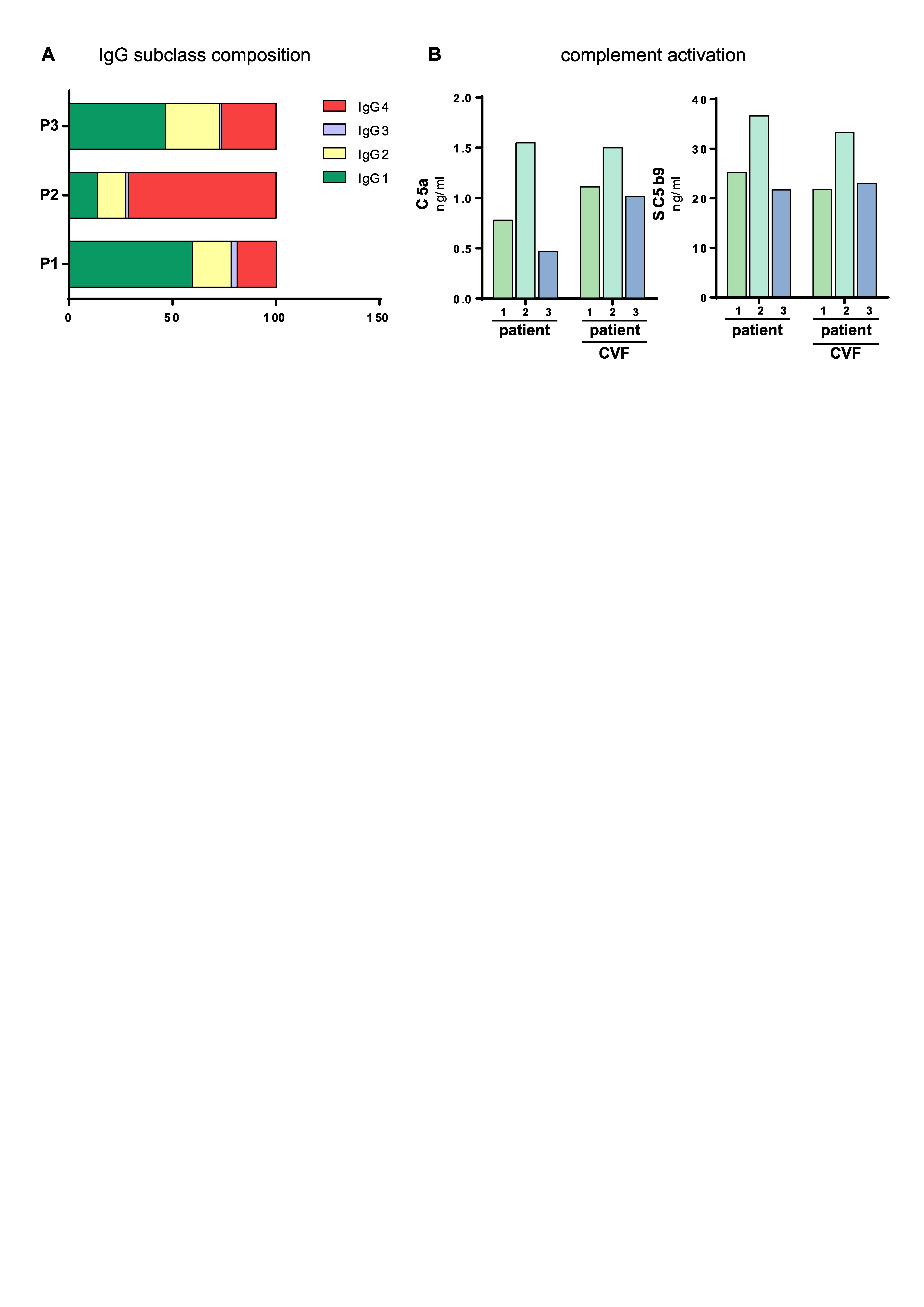
**

**Supplementary Figure 1.**

(**A**) Serum samples of three IgLON5 patients were incubated on a IgLON5 coated Mosaik plate and subsequently stained with antibodies against IgG1-4. Dilutions for titer determination led to semiquantitative analysis. (**B**) Multiplex analysis of complement activation was performed on iNeurons incubated with either solely IgG fraction or IgG fraction and the complement inhibitor cobra venom factor (CVF).

**
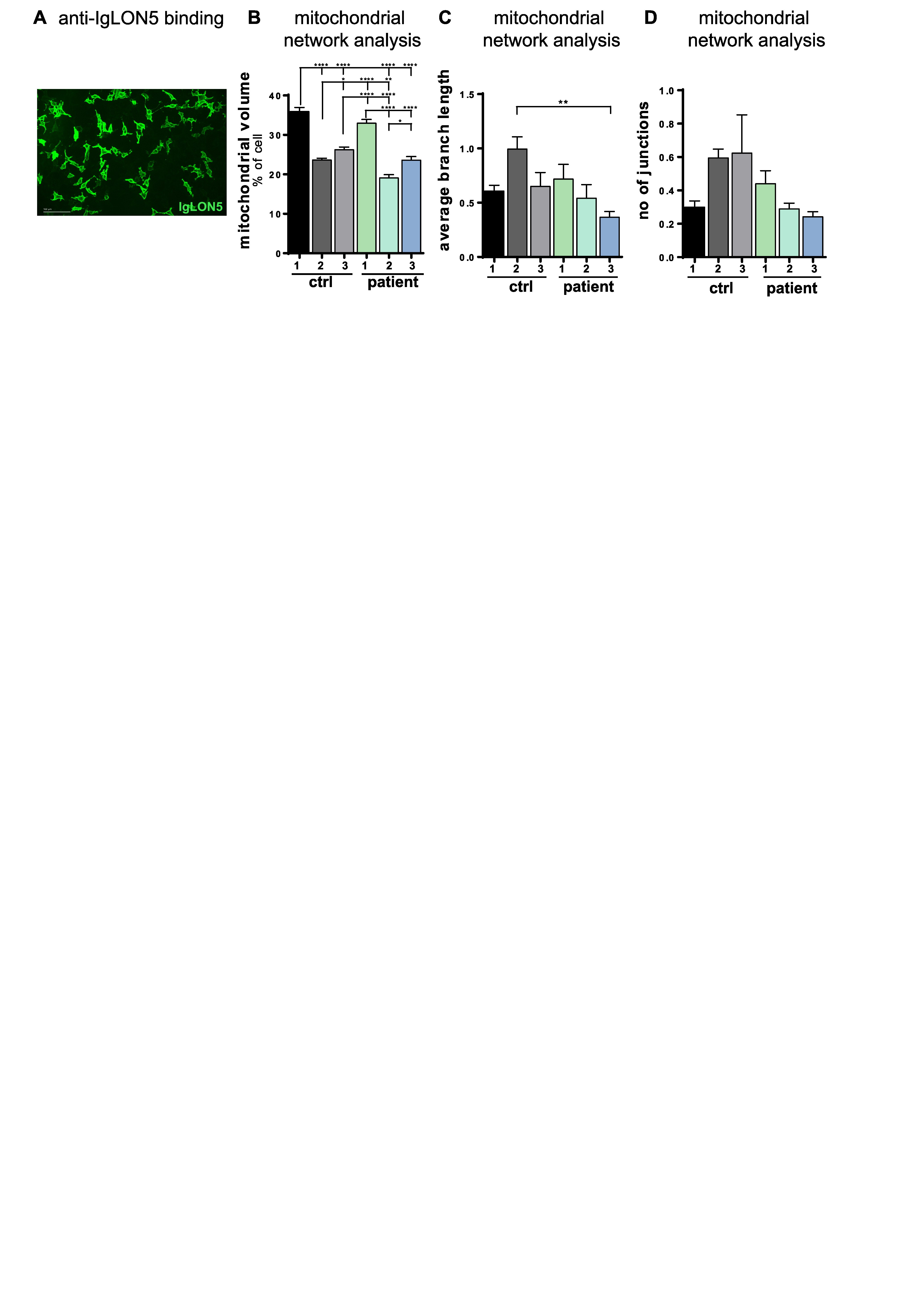
**

**Supplementary Figure 2.**

(**A**) HEK cells transfected with IgLON5 and incubated with patient IgG. (**B-D**) Mitochondrial
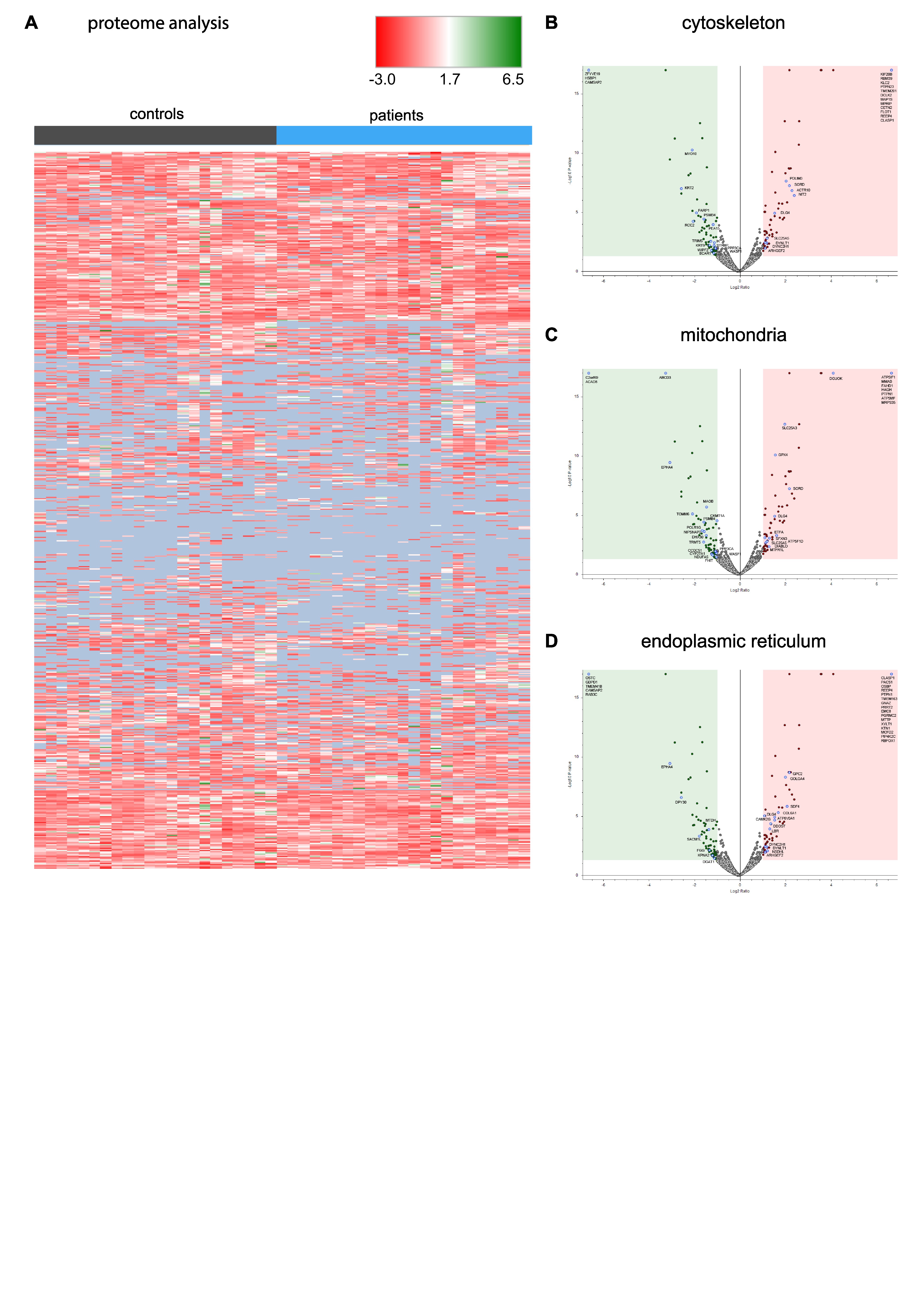
network analyses of iNeurons stained with MitoTracker**.**

**Supplementary Figure 3.**

(**A**) Cluster analysis of differentially expressed proteins among iNeurons treated with patient IgG or control IgG performed as label free quantification (LFQ) of high-resolution mass spectrometry data. (**B-D**) Volcano plots of differentially expressed proteins in patient IgG treated iNeurons vs. control IgG treated iNeurons assigned to the GO terms cytoskeleton (**B**), mitochondria (**C**), or endoplasmic reticulum (**D**).


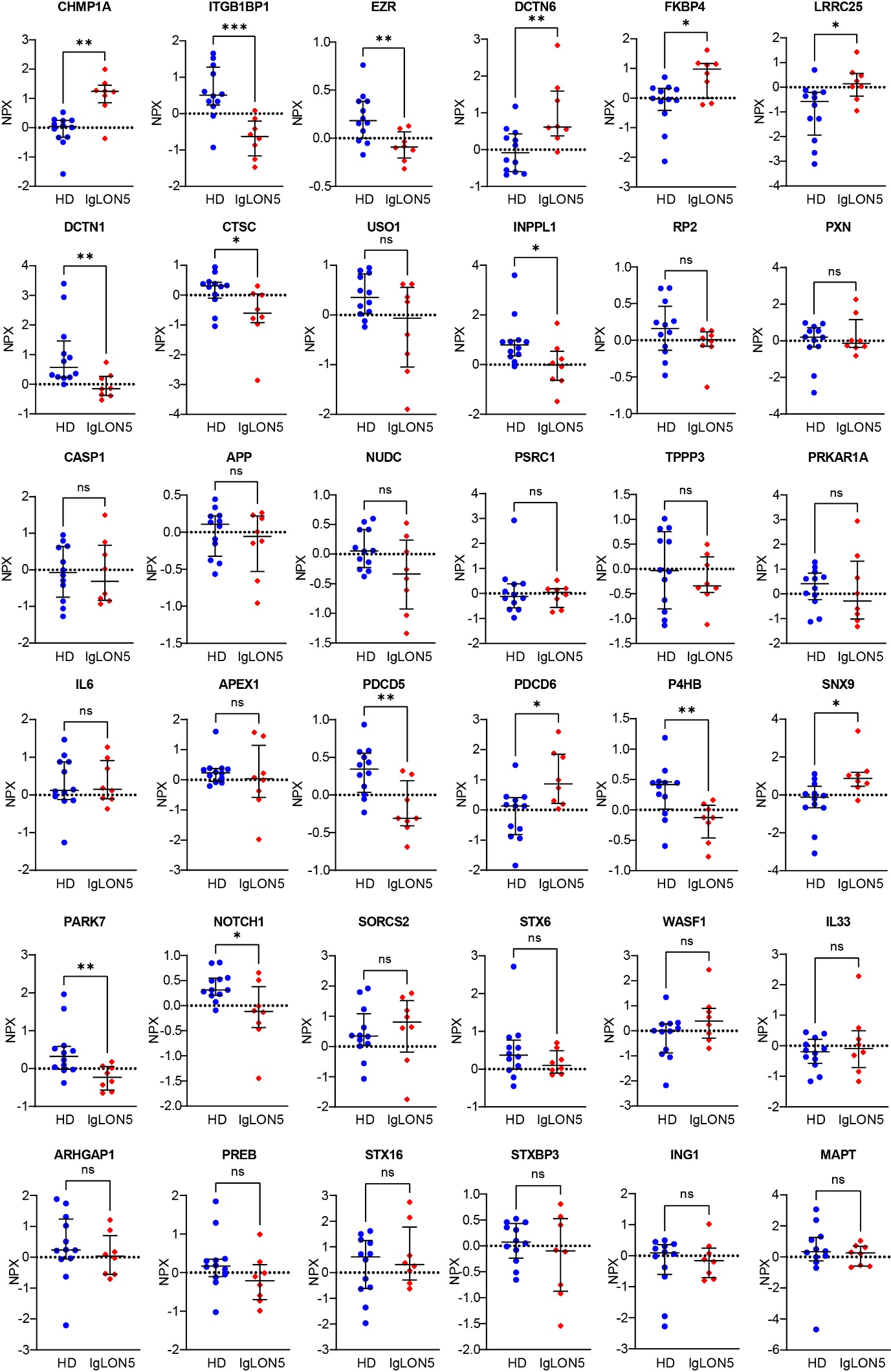


**Supplementary Figure 4.**

Donor-individual NPX values of serum proteins analysed by OLink PEA-NGS and identified in Fig. 3. Statistical analysis was performed by Mann-Whitney test, ns p > 0.05, *p < 0.05, **p < 0.01, ***p < 0.001, **** p < 0.0001.

**Supplementary Table 1. Clinical characteristics of anti-IgLON5 patients.**

| **Subject** | **Age at sampling, y** | **Disease duration, y** | **Phenotype** | | | | | |
| --- | --- | --- | --- | --- | --- | --- | --- | --- |
|  |  |  | **Presenting complaint** | **Sleep disorder** | **Bulbar syndrome** | **PSP like** | **Cognitive impairment** | **Overall phenotype** |
| Patient 1 | 68 | 5 | Sleep disorder | +++ | - | + | ++ | Sleep disorder |
| Patient 2 | 64 | 3 | Dysphagia,  sleep disorder | ++ | ++ | + | - | Sleep + bulbar |
| Patient 3 | 52 | 1 | Dysphagia | - | +++ | + | - | Bulbar |
| Patient 4* | 72 | 8 | Dysphagia | (+) | +++ | + | + | Bulbar |
| Patient 5* | 76 | 2 | Dysphagia | + | +++ | - | ++ | Bulbar |
| Patient 6* | 81 | 7 | Polyneuropathy/  Neuromyotonia | ++ | - | +++ | ++ | PSP like / Sleep Disorder |
| Patient 7* | 57 | 2 | Laryngeal spasms | ++ | +++ | + | - | Bulbar > sleep disorder > PSP-like |
| Patient 8* | 51 | 3 | Dysarthria, sleep disorder | +++ | +++ | - | + | Bulbar |
| Ctrl 1 | 55 | N/A |  |  |  |  |  |  |
| Ctrl 2 | 79 | N/A |  |  |  |  |  |  |
| Ctrl 3 | 60 | N/A |  |  |  |  |  |  |

Symptom presentation: - not present; (+) very mild, + mild; ++ moderate; +++ severe; y year(s); N/A: not applicable; * note that patient 4-8 were only analysed with OLink.
